# A method, framework, and tutorial for efficiently simulating models of decision-making

**DOI:** 10.3758/s13428-019-01219-z

**Published:** 2019-03-28

**Authors:** Nathan J. Evans

**Affiliations:** 1 Department of Psychology, University of Amsterdam, Amsterdam, The Netherlands; 2 Department of Psychology, Vanderbilt University, Nashville, TN USA

**Keywords:** Decision-making, Evidence accumulation models, Random number generation, Probability density approximation

## Abstract

Evidence accumulation models (EAMs) have become the dominant models of rapid decision-making. Several variants of these models have been proposed, ranging from the simple linear ballistic accumulator (LBA) to the more complex leaky-competing accumulator (LCA), and further extensions that include time-varying rates of evidence accumulation or decision thresholds. Although applications of the simpler variants have been widespread, applications of the more complex models have been fewer, largely due to their intractable likelihood function and the computational cost of mass simulation. Here, I present a framework for efficiently fitting complex EAMs, which uses a new, efficient method of simulating these models. I find that the majority of simulation time is taken up by random number generation (RNG) from the normal distribution, needed for the stochastic noise of the differential equation. To reduce this inefficiency, I propose using the well-known concept within computer science of “look-up tables” (LUTs) as an approximation to the inverse cumulative density function (iCDF) method of RNG, which I call “LUT-iCDF”. I show that when using an appropriately sized LUT, simulations using LUT-iCDF closely match those from the standard RNG method in R. My framework, which I provide a detailed tutorial on how to implement, includes C code for *12* different variants of EAMs using the LUT-iCDF method, and should make the implementation of complex EAMs easier and faster.

## Introduction

Evidence accumulation models (EAMs; Stone 1960) are currently the dominant modeling framework within rapid decision-making, having aided our understanding of stop-signal paradigms (Matzke, Dolan, Logan, Brown, & Wagenmakers, 2013), absolute identification (Brown, Marley, Donkin, & Heathcote, 2008), performance optimality (Starns & Ratcliff, 2012; Evans & Brown, 2017; Evans, Bennett, & Brown, 2018), clinical populations (Ho et al., 2014), performance improvement over practice (Evans, Brown, Mewhort, & Heathcote, 2018), more complex decisions (Hawkins et al., 2014), and having provided links to other types of data, such as personality measures (Evans, Rae, Bushmakin, Rubin, & Brown, 2017), genetic information (Evans, Steyvers, & Brown, 2018), and neural recordings (Forstmann et al., 2011). EAMs propose that decisions are made through a process where evidence accumulates for each of the different decision alternatives at some rate (known as the “drift rate”) until the evidence for one of these alternatives reaches some threshold level of evidence (known as the “decision threshold”), where a response is triggered. Importantly, EAMs are able to account for the well-known speed–accuracy tradeoff (SAT) by analyzing response time and accuracy data in unison, and estimate theoretically meaningful parameters from the observed response time distributions (Donkin, Averell, Brown, & Heathcote, 2009).

Although all EAMs contain the general process described above, each EAM differs in the specifics of the proposed process. Where some EAMs have attempted to provide a parsimonious model suitable for easy application (e.g., the linear ballistic accumulation [LBA; Brown & Heathcote 2008]), several other EAMs have attempted to provide a more process-focused model definition. For example, the leaky-competing accumulator (LCA; Usher & McClelland 2001) contains a process based upon what would be expected from underlying neural architecture, derived from findings within the neuroscience and neurophysiology literature (e.g., Rumelhart, Hinton, & McClelland, 1986; Softky & Koch, 1993; Shadlen & Newsome, 1994; Amit & Tsodyks, 1991; Chelazzi, Miller, Duncan, & Desimone, 1993). Beyond the LCA, several new proposals have incorporated time-varying drift rates (Servant, Montagnini, & Burle, 2014; Evans, Hawkins, Boehm, Wagenmakers, & Brown, 2017)or decision thresholds (Hawkins, Forstmann, Wagenmakers, Ratcliff, & Brown, 2015), where the values of these parameters systematically vary across the course of the trial. These proposals have included “urgency signals” that are applied to the accumulated evidence to prevent overly slow responses (Cisek, Puskas, & El-Murr, 2009; Thura, Beauregard-Racine, Fradet, & Cisek, 2012), piecewise models to account for objectively changing evidence over the course of the decision (Holmes, Trueblood, & Heathcote, 2016; Holmes & Trueblood, 2018), the use of single-cell recording data as direct input for the drift rates (Purcell et al., 2010), and decreases in the amount of evidence required to trigger a decision over time (i.e., collapsing thresholds; Ditterich, 2006; Drugowitsch, Moreno-Bote, Churchland, Shadlen, & Pouget, 2012).

However, implementing these more complex EAMs often comes at a practical cost, where the probability density functions (PDFs) required to fit the models are either unknown, or computationally burdensome to implement. In contrast, the simple functional form of the LBA results in an analytically solvable PDF, which has helped the LBA become a useful tool for researchers in decision-making (e.g., Evans, Rae, Bushmakin, Rubin, & Brown, 2017; Ho et al., 2014; Brown, Marley, Donkin, & Heathcote, 2008; Donkin, Averell, Brown, & Heathcote, 2009; Holmes, Trueblood, & Heathcote, 2016; Evans, Steyvers, & Brown, 2018). Applications of models that contain unknown or intractable PDFs have relied on methods that involve mass simulation. Initially, these methods involved either “hand-tuning” of parameter values (Thura, Beauregard-Racine, Fradet, & Cisek, 2012), small grid-based searches (Tsetsos, Usher, & Chater, 2010), or minimization routines (Hawkins, Wagenmakers, Ratcliff, & Brown, 2015; Evans et al., 2017), with the best-fitting parameters being determined by the smallest discrepancy between the data and the model predictions on some summary statistic (e.g., *χ*^2^). More recent implementations have involved the use of pseudo-likelihood methods, such as probability density approximation (PDA; Turner & Sederberg 2014; Holmes 2015), which involve fitting a density kernel to the model predictions generated through simulation, allowing a simulation-based PDF to be obtained for the model (Turner, Schley, Muller, & Tsetsos, 2018). PDA provides several benefits over the minimization methods, allowing the models to be fit with likelihood-based methods of estimation, such as Bayesian methods that allow for more complete methods of selecting between competing models (Evans, Howard, Heathcote, & Brown, 2017; Evans & Brown, 2018; Gronau et al., 2017). However, pseudo-likelihood methods also require mass simulation, with a large number of simulated trials being required to ensure an accurate estimate of the PDF. Unfortunately, this can result in more complex EAMs being practically impossible to fit, due to the large amount of time taken up by mass simulation.

Here, I present a framework for efficiently fitting complex EAMs, which uses a new, efficient method of simulating these models. My method, which I call *LUT-iCDF*, uses the well-known concept within computer science of “look-up tables” (LUTs) to provide a fast approximation of the inverse cumulative density function (iCDF) method of random number generation (RNG) for the normal distribution, which will be explained in more detail in the next section “The LUT-iCDF method”. My framework includes C code for simulating *12* different variants and sub-variants of EAMs, R wrappers that make the C code easy to use, and a basic implementation of PDA in R to allow these models to be fit in maximum likelihood or Bayesian frameworks (though the C code and R wrappers can be used within any simulation-based fitting framework, such as *χ*^2^). My method of RNG is also generalizable to any model where RNG from the normal distribution is a large time cost for the simulations (e.g., any stochastic differential equation). Within this article, I provide a full description and testing of my LUT-iCDF method of RNG from the normal distribution, a tutorial on how to implement the included code, and a brief description of the models included (with references to where further details can be found). Overall, the aim of this article is to make the implementation of complex EAMs easier and faster, in an attempt to increase their usage.

## The LUT-iCDF method

In this section, I detail my proposed method for fast RNG from the normal distribution. Specifically, I show the large computational cost of RNG from the normal distribution. From there, I explain my method and show the associated speed increase in simulation time. Lastly, I discuss some theoretical limitations of my method, but show that in practical applications my method leads to identical results as the standard RNG function in R.

The programming language C has been used to provide faster performance in some previous applications of complex EAMs (e.g., Hawkins, Wagenmakers, Ratcliff, & Brown, 2015; Evans, Hawkins, Boehm, Wagenmakers, & Brown, 2017; Voss & Voss, 2007). Specifically, C is a programming language that underlies several mainstream data analysis programming languages, such as R (R Core Team, 2014) and MATLAB. In general, C implementations can be faster than those of mainstream data analysis programming languages, but also harder to create. However, the simulation process for most EAMs can be performed within a short amount of code using basic mathematical functions, making them fairly simple to implement in C with large potential increases in computation speed. An example of these speed increases for each of the models in my framework can be seen in Table 1. In all cases, implementing the model in C (column 4) is *at least* twice as fast as implementing the model in R (column 2).

**Table 1:** Displays the mean (standard deviation) computation time over 100 independent runs to simulate each of the models in my framework (rows) for 10,000 trials of 200 time-steps each

Model	R code	R code LUT	C code	C code LUT
DIFF	0.383 (0.066)	0.317 (0.069)	0.165 (0.008)	0.027 (0.002)
DIFF P	0.391 (0.068)	0.324 (0.067)	0.163 (0.007)	0.027 (0.002)
DIFF TV	0.359 (0.063)	0.303 (0.069)	0.163 (0.006)	0.028 (0.002)
DIFF DB	0.41 (0.071)	0.333 (0.065)	0.164 (0.007)	0.028 (0.002)
LCA	0.855 (0.068)	0.768 (0.056)	0.344 (0.012)	0.058 (0.006)
LCA P	0.864 (0.091)	0.722 (0.042)	0.346 (0.012)	0.062 (0.006)
LCA TV	0.92 (0.032)	0.807 (0.069)	0.345 (0.011)	0.06 (0.005)
LCA DB	0.835 (0.055)	0.741 (0.059)	0.352 (0.035)	0.061 (0.006)
UGM	0.42 (0.078)	0.358 (0.073)	0.18 (0.008)	0.043 (0.003)
UGM TV	0.387 (0.07)	0.324 (0.069)	0.179 (0.008)	0.043 (0.003)
LBA	0.041 (0.024)	0.043 (0.025)	0.002 (0.001)	0.001 (0.001)
LBA P	0.055 (0.027)	0.057 (0.031)	0.005 (0.001)	0.003 (0.001)

Columns display the names of the models, and different methods of simulation: R code using the “rnorm” function, R code using the LUT-iCDF method, C code using the “norm_rand()” function, and C code using the LUT-iCDF method. In all cases, the LUT-iCDF method was implemented with a granularity of 0.0001. Within the table, “DIFF” refers to the diffusion model, “P” refers to a piecewise extension, “TV” refers to a time-varying drift rate extension, and “DB” refers to a time-varying threshold extension. Note that all of these timing benchmarks are based on my computer, and will differ between different hardware and software. However, these benchmarks serve as an example of the relative speed-up gained using the LUT-iCDF method

However, one commonly overlooked aspect that can have a large impact on simulation speed is the method of RNG. Importantly, for all EAMs that contain stochastic, within-trial noise (i.e., every EAM except the LBA), RNG from the normal distribution needs to be performed on every time-step of every simulated trial. Therefore, if the method of RNG is inefficient, it will have detrimental effects on the overall speed of the implementation. For example, when simulating the diffusion model (Ratcliff, 1978)—one of the most commonly used EAMS—using C’s “norm_rand()” function for the RNG of the stochastic noise, simulating 10,000 trials that each contain 200 time-steps takes an average of 165 ms (SD = 8 ms). However, when only running the deterministic parts of the code (i.e., setting the stochastic noise to a fixed value), the simulation takes an average of only 6 ms (SD = 1 ms), meaning that the RNG through “norm_rand()” is taking up more than 96% of the simulation time. Another RNG method that is easy to implement is the inverse cumulative density function (iCDF), which requires generating random uniform numbers between 0 and 1 (i.e., *U*[0,1]) and taking their iCDF under the standard normal distribution. However, the simulation^1^ still takes an average of 122 ms (SD = 5 ms), meaning that over 95% of the simulation time is still being taken up by RNG.

To reduce this inefficiency, I propose using a look-up table (LUT)—a concept from computer science—to approximate the iCDF method of RNG, which I call *LUT-iCDF*. LUTs are commonly used when specific calculations are computationally costly relative to other elements of the code, and these calculations have to be performed repeatedly throughout the process. LUTs map “before calculation” values to their respective “after calculation” values, meaning that these computationally taxing calculations only need to be performed once. A classic example of the use of LUTs is the calculation of *p* values before advancements in computing made integrating the tails of these distributions trivial, where the test statistic and degrees of freedom were “looked-up” within a table to find the respective *p* value. However, my LUT-iCDF method is also able to remove the inefficiency of having to search the LUT for the matching before calculation element (i.e., the “looking-up” process) by converting the uniformly distributed numbers (i.e., *U*[0,1]) to uniformly distributed integers that match the size of the table (i.e., *U*[1,*N*], where *N* is the number of elements in the LUT). Provided that the LUT corresponds to symmetric and equally spaced elements from the *U*[0,1], which I describe how to create below, the randomly generated integers can be directly used as randomly generated indexes of the LUT, and the LUT element corresponding to the index is a random sample from the standard normal distribution.

**Fig. 1:**
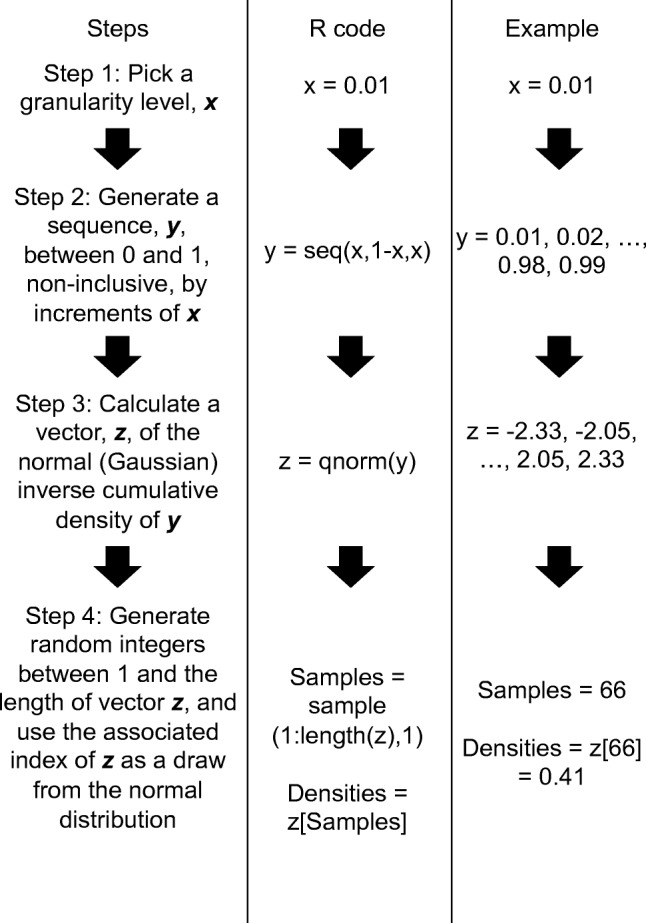
A flowchart of my proposed look-up table approximation to the inverse cumulative density function method (LUT-iCDF) of random number generation from the normal distribution. The columns, from left to right, show the written steps, the associated R code, and an example

Implementation of the LUT-iCDF method requires four simple steps, which can be seen as a flowchart in Fig. 1. The process begins by generating a sequence of symmetrical (i.e., centered on 0.5), equally spaced numbers between 0 and 1 (non-inclusive, as 0 and 1 reflect −*∞* and *∞* of the standard normal, respectively), which creates an unbiased approximation of a uniform distribution between 0 and 1. An easy way to do this is to generate a sequence of numbers with granularity (i.e., spacing) *x*, which start at 0 + *x* and finish at 1 − *x*, as shown in Step 1 and Step 2 of Fig. 1. Next, the iCDF is calculated for each number in the sequence, and these iCDF values are stored in the LUT, which contains $\frac {1 - x}{x}$ elements (Step 3). Lastly, random uniformly distributed integers are generated that have a minimum value of 1 and a maximum value of the total number of elements in the LUT, and the integers form a random index of the LUT, with the corresponding elements of the LUT being samples from the standard normal distribution (Step 4). As shown in Table 1, the implementation of the LUT-iCDF method greatly increases simulation speed in both R (column 3) and C (column 5) code, with the simulation of the diffusion model for 10,000 trials of 200 time-steps only taking 27 ms (SD = 2 ms), meaning that RNG is taking up approximately 78% of the time, in contrast to the 95%+ of standard methods.

### Assessing the LUT-iCDF approximation accuracy

As mentioned above, the LUT-iCDF method involves some level of approximation, and therefore, could potentially lead to inaccuracies. Importantly, the accuracy of the approximation to the normal distribution is dependent on the granularity of the sequence that approximates the *U*[0,1], meaning that a small granularity should be used to create a large number of points in the LUT-iCDF approximation. Specifically, granularities that are too large will result in “truncations” of the approximated normal distribution, where values from certain parts of the distribution are never sampled, such as the tails. However, granularities that are too small will result in the LUT becoming large, and sufficiently large LUTs may cause increases in computation time.

**Fig. 2:**
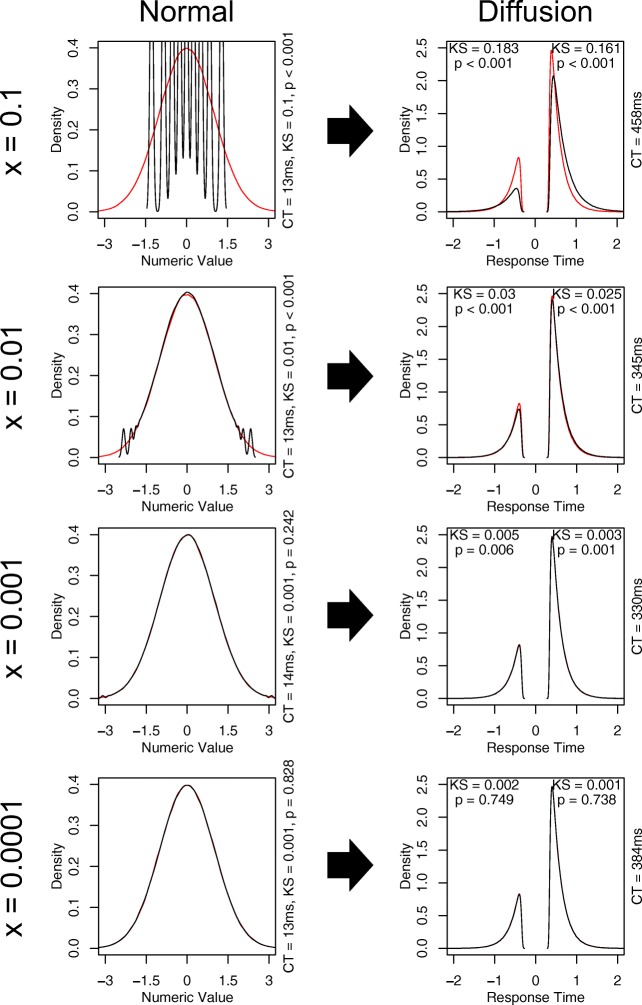
Displays a comparison between the “rnorm” function in R (*red*) and a C implementation of my LUT-iCDF method (black) for 1,000,000 samples from the normal distribution (*left column*) and 1,000,000 simulated trials of the diffusion model (*right column*), for different granularities of the LUT-iCDF (*rows*). For the diffusion model simulations, response time distributions corresponding to responses for the second alternative are plotted as negative values on the *x*-axis, to make the distributions for different responses more easily distinguished. In all plots, “CT” refers to the computation time of taking the 1,000,000 samples, “KS” refers to the test statistic of the non-parametric Kolmogorov–Smirnov test for equivalent distributions, and “*p*” refers to the *p* value for the Kolmogorov–Smirnov test

Figure 2 provides an assessment of the accuracy of different LUT-iCDF granularities (different rows) in approximating the standard normal distribution (left column), and how inaccuracies in the approximation of the standard normal distribution influence the approximation of the diffusion process (right column). For the smallest granularity (x = 0.1; top row) the LUT-iCDF produces a poor approximation of the normal distribution, with the samples forming nine separate spikes at the nine different values in the LUT. The poor approximation of the normal distribution is reflected in the diffusion process, as the LUT-iCDF simulation produces responses that are more likely to be slower and correct than when simulating using the actual normal distribution. The second granularity (x = 0.01; second row) provides an improvement, with the LUT-iCDF producing a good approximation of the normal distribution in most regions. However, the approximation becomes poorer in the tails, showing behavior similar to the previous granularity and resulting in the Kolmogorov–Smirnov (KS) test suggesting that the distributions differ from one another (KS = 0.01, *p* < 0.001), which also continues to influence the diffusion process (correct responses: KS = 0.025, *p* < 0.001; incorrect responses: KS = 0.03, *p* < 0.001). The third granularity (x = 0.001; third row) provides a very close approximation to the normal distribution, with only the tails of the LUT-iCDF approximated distribution (i.e., SD > 3) showing any noticeable deviations from the normal distribution, and the KS test failing to suggest that there are any differences between the distributions (KS = 0.001, *p* = 0.242). However, these minor deviations appear to still cause deviations in the predictions of the diffusion process: although the simulation of the diffusion process using the LUT-iCDF and the actual normal distribution are difficult to visually distinguish between, the KS test continues to suggest that these distributions differ from one another (correct responses: KS = 0.003, *p* = 0.001; incorrect responses: KS = 0.005, *p* = 0.006).

**Fig. 3:**
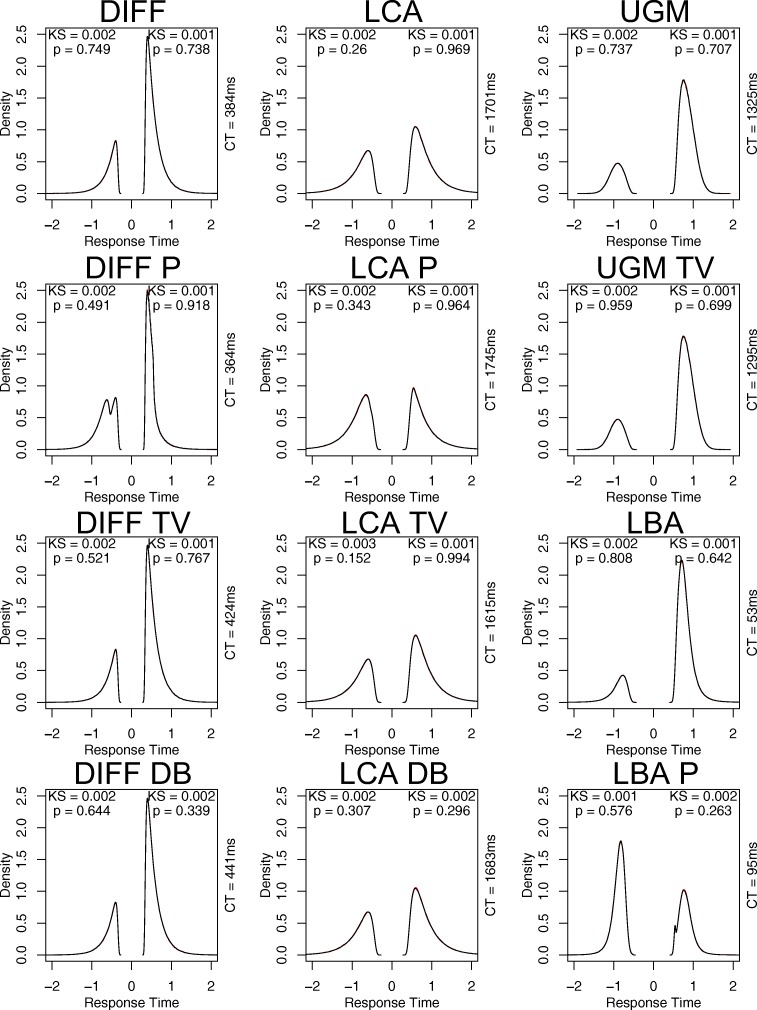
Displays a comparison between the “rnorm” function in R (*red*) and a C implementation of my LUT-iCDF method with granularity 0.0001 (*black*) for 1,000,000 simulated trials of each of the models in my framework (different panels). Response time distributions corresponding to responses for the second alternative are plotted as negative values on the *x*-axis, to make the distributions for different responses more easily distinguished. In all plots, “CT” refers to the computation time of taking the 1,000,000 samples, “KS” refers to the test statistic of the non-parametric Kolmogorov–Smirnov test for equivalent distributions, “*p*” refers to the *p* value for the Kolmogorov–Smirnov test, “DIFF” refers to the diffusion model, “P” refers to a piecewise extension, “TV” refers to a time-varying drift rate extension, and “DB” refers to a time-varying threshold extension

For the largest granularity included (x = 0.0001; bottom row) the LUT-iCDF appears to provide a near identical approximation to the normal distribution, both visually and in terms of the KS test (KS = 0.001, *p* = 0.828). This accurate approximation also carries over to the simulation of the diffusion process, with the distributions for correct (KS = 0.001, *p* = 0.738) and incorrect (KS = 0.002, *p* = 0.749) responses generated by the LUT-iCDF approximation being near identical to using the actual normal distribution, both visually and in terms of the KS test. Based on this assessment, it appears that the LUT-iCDF method a granularity of 0.0001 (i.e., 10^− 4^) provides an accurate approximation of the diffusion process, and that RNG from a LUT of this size (i.e., 9999 elements) is approximately as quick as generating with the smallest LUT assessed (nine elements; both took approximately 13 ms to generate 1,000,000 normally distributed samples). Although the granularity could potentially be made even smaller in an attempt ensure the accuracy of the approximation, further decreases (i.e., x = 0.00001) began to result in large increases in computation time (normal = 19 ms, diffusion = 603 ms), which I discuss further in the next sub-section on LUT augmentations. A further assessment of the approximation accuracy of the LUT-iCDF method with a granularity of 0.0001 can be seen in Fig. 3, which compares the simulation of each of the models in my framework (which will each be discussed in detail in the “Models” section) with LUT-iCDF to those using the actual normal distribution. In all cases, the LUT-iCDF with 0.0001 granularity appears to provide a near identical approximation, both visually and according to KS tests, suggesting that this granularity generally appears to provide an accurate approximation of the normal distribution and the simulation of EAMs. Therefore, I recommend the use of the 0.0001 granularity (i.e., 9999 table elements), which I implement in all of my included code; however, the granularity can be easily changed within the code, which I explain in the “Implementation” section.

### Potential LUT augmentations

As discussed previously, my LUT-iCDF method is able to remove the inefficiency of searching the LUT by using uniformly distributed integers that match the size of the table. This requires that the elements of the LUT correspond to the iCDF of symmetric and equally spaced elements from the *U*[0,1], which I implement in all of my code. However, there may be situations where using a non-symmetric and/or non-equally spaced LUT may be more efficient, as removing these constraints may allow the LUT to be reduced in size. Here I briefly explore two of these possibilities, and assess under what conditions they would provide faster sampling from the normal distribution.

One possibility to reduce the size of the LUT would be to only use the elements corresponding to the positive values of the normal distribution. As each half of the LUT provides duplicate information in absolute magnitude (e.g., the iCDF of 0.6 is 0.253, and the iCDF of 0.4 is -0.253), the size of the LUT could be halved without any loss of information. However, this would mean that the random uniform integer would not directly correspond to an index of the LUT, meaning that a few extra lines of code, including an “if” statement, which can be computationally expensive, need to be added. A comparison of computation time for the standard LUT-iCDF (column 2) and the halved version (column 3) for different granularities can be seen in Table 2. Interestingly, when the LUT is small, the extra lines of code required for the halved version cause it to sample more slowly than the standard LUT-iCDF method. However, as the granularity is decreased, this discrepancy also decreases, until the halved version becomes faster than the standard LUT-iCDF (x = 10^− 7^). Therefore, the halved LUT may provide a more efficient LUT in situations where a fine-grained approximation is required.

**Table 2:** Displays a comparison in computation time for generating 1,000,000 samples from the normal distribution between the LUT-iCDF method, the version with a halved LUT (“Halved”), and the version that requires directly mapping to before calculation values to their closest matching after calculation value (“Direct mapping”) for different granularities (rows)

Granularity	LUT-iCDF	Halved	Direct mapping
0.1	0.013	0.022	0.028
0.01	0.013	0.021	0.065
0.001	0.014	0.021	0.425
0.0001	0.013	0.022	3.955
10^− 5^	0.019	0.023	40.026
10^− 6^	0.035	0.041	
10^− 7^	0.072	0.058	

Note that the final two rows were not calculated for the “Direct mapping” version, due to the large computation time

Another possibility to reduce the size of the LUT would be to create a standard LUT, which requires directly mapping to before-calculation values to their closest matching after-calculation value. This would allow the LUT to be both non-symmetric and have variable spacing between elements, which could allow parts of the table that provide little information (e.g., where elements have near identical values) to be removed. However, this would also require the implementation of a search algorithm to find the after-calculation value that provides the closest match to the before-calculation value, which could result in additional computational overheads. A comparison of computation time for the standard LUT-iCDF (column 2) and the directly mapping version (column 4) for different granularities can be seen in Table 2. Interestingly, the added computational time required to search the LUT is much greater than the other parts of the process, meaning that the size of the LUT would need to be greatly reduced (e.g., 9,999,999 elements to 99 elements) to make the direct mapping version faster than the LUT-iCDF (or, alternatively, a much more efficient search algorithm would need to be used).

## Implementation

In this section, I provide a tutorial on how to implement the code within my framework. Specifically, I explain how to use and call the C code within R. After that, I detail the R simulation wrappers that I have included to call the C code, which should provide users with an easier method of implementation than directly calling the C code. I also provide a brief worked example on how to use the code through simulating the LBA. Lastly, I discuss the PDA code that I have included, and the likelihood function code that combines the simulations and PDA method to produce a pseudo-likelihood approximation for the model. Based on this tutorial, interested readers should be able to implement my simulation code (and my PDA code, where required) into their current fitting methods, giving them the ability to efficiently fit any of these complex EAMs.

### Compiling and loading the C code

Here, I explain how to implement the C code within R. Tables 3 and 4 provide most of the key details on the C code included in my framework. Table 3 provides the different variables that are passed into the C code and the different models that require them, and Table 4 displays which code files are associated with which models. Importantly, when a variable is labeled as an integer, it must be passed to the C code as an integer (i.e., “as.integer(VARIABLE)”), and when it is labeled as a double, it must be passed to the C code as a double. Failing to make each variable the correct type will result in the C code either crashing R, or returning incorrect results. An example of how to call the C code for each model can be seen within the R simulation code wrappers.

**Table 3:** Displays the input variables for the C code in my framework. The input variable “parameters” refers to the multitude of variables that are the unique to each model

Variable name	Variable type	Description
nRespAlt	integer	The number of response alternatives. Only for models with separate accumulation rates
n	double	The number of trials to be simulated
resp	double	A vector of length “n” that is entered as 0’s and returned as a number corresponding to the response alternative chosen
rt	double	A vector of length “n” that is entered as 0’s and returned as the response time in seconds
h	double	The time-step used. Only for models with within-trial noise
maxiter	double	The maximum number of steps run performing terminating the with within-trial noise
swapTime	double	The time at which the change of evidence occurs within the model. Only for piecewise models
rangeLow	integer	The lowest possible index of the look-up table (always 0)
rangeHigh	integer	The highest possible index of the look-up table (number of elements of the table minus 1)
randomTable	double	A vector that contains the iCDF LUT
“parameters”	double	Multiple variables, which are unique to each model. All parameters need to be entered as type “double”

**Table 4:** Displays the different models included within my framework (rows), and the relevant C code file, R code wrapper file, and PDA code (in R) file

Model	C code	R wrapper	PDA code
LBA	lba.c	simulate-lba.R	PDA.R
LBA piecewise	plba.c	simulate-plba.R	PDA_p.R
Diffusion	DIFF.c	simulate-DIFF.R	PDA.R
Diffusion piecewise	pDIFF.c	simulate-pDIFF.R	PDA_p.R
Diffusion time-varying drift rate	DIFF-tv.c	simulate-DIFF-tv.R	PDA_tv.R
Diffusion time-varying thresholds	DIFF-db.c	simulate-DIFF-db.R	PDA_db.R
LCA	lca.c	simulate-lca.R	PDA.R
LCA piecewise	plca.c	simulate-plca.R	PDA_p.R
LCA time-varying drift rate	lca-tv.c	simulate-lca-tv.R	PDA_tv.R
LCA time-varying thresholds	lca-db.c	simulate-lca-db.R	PDA_db.R
UGM	ugm.c	simulate-ugm.R	PDA.R
UGM time-varying drift rate	ugm-tv.c	simulate-ugm-tv.R	PDA_tv.R

A few things need to be set up before using the C code. Firstly, the code must be compiled, which is a relatively easy process in R. Specifically, the C code can be compiled by typing “system(‘R CMD SHLIB *codeName*.c’)” into the R console while in the same working directory as the C code, which will create a compiled file. The extension of the compiled file will differ based on the compiler and operating system. For Mac OS and Linux, the C code is usually compiled into a “.so” file, and for Windows a “.dll” file. All of the code within my framework assumes that the compiled file has a “.so” extension, but this can be easily changed for users where the code is compiled into another type of file. Once the code is compiled, the compiled file needs to be loaded into the R environment, which can be done with “dyn.load(‘*codeName*.so’)”. Examples of these steps can be seen within any of the “simulate” R files that call the C code for simulation.

### Using the R wrappers to call the C code

Next, I detail the R wrappers that I have included for simulating the models and how to use them. Table 5 provides the variables that are passed into the main function in the R wrapper for each model, and Table 4 provides the file that contain the R wrapper for simulating each model. An example of how to call the R wrappers for each model can be seen within the example PDA pseudo-likelihood functions that I have included.

**Table 5:** Displays the input variables for the R wrapper code in my framework, and which classes of models the input variables are applicable for

Variable name	Variable type	Description
N	double	The number of trials to be simulated
params	double	A vector of named parameters for the model to be simulated
v	double	A vector (or in the case of the LCA, a matrix) of the drift rate for each time step of the simulation. Only for time-varying drift rate models
aU	double	A vector of the upper threshold for each time step of the simulation. Only for time-varying threshold models
aL	double	A vector of the lower threshold for each time step of the simulation. Only for time-varying threshold models
stepSize	double	The time-step used
swapTime	double	The time at which the change of evidence occurs within the model. Only for piecewise models
maxCounter	double	The maximum number of steps run performing terminating the simulated trial
n.table.options	integer	The number of elements in the LUT
use.table	double	A vector that contains the iCDF LUT

The elements that are passed into these R wrappers can be placed into three general categories: simulation requirements, stochastic differential equation requirements, and LUT requirements. The simulation requirements are the basic variables required to run the simulation, which for all models include the variable “N”, and the vector “params”. Importantly, the “params” vector needs to be a vector of *named* parameter values, with the names being specific to the model being implemented. The stochastic differential equation requirement are for all models within this class (i.e., every model except from the LBA and pLBA), and are the “maxCounter” and “stepSize” variables. By convention, the time-step is defined in seconds, meaning that a time-step of 1 ms should be entered as 0.001. Lastly, the LUT requirements are those needed for the LUT-iCDF method, and are the vector “use.table” and the variable “n.table.options”.

It is also important to note that there are some key differences between the R wrappers of the different broad classes of models. In addition to the input variables discussed above, the piecewise and time-varying models each require additional input variables. The piecewise models require the variable “swapTime”, which is the time (in seconds) when the model switches to the “after evidence change” drift rates. Piecewise models also require the “params” vector to contain drift rates for both before and after the change in. The time-varying drift-rate models no longer include the drift rate parameter(s) in the “params” vector, which are instead contained in the input vector “v”, which is “maxCounter” in length and contains the drift rate for each time-step. For the LCA, “v” is instead a matrix, where the rows are the drift rates for each time-step and the columns are the different response alternatives. The time-varying threshold models no longer include the threshold parameter(s) in the “params” vector, which are instead contained in the input vectors “aU” and “aL”, which are “maxCounter” in length and contain the upper and lower thresholds for each step, respectively. For the LCA, the time-varying threshold is instead a matrix called “a”, where the rows are the thresholds for each time-step and the columns are the different response alternatives.

In general, the contents of the main function in each R wrapper is relatively simple and mostly involves re-arranging variables into an appropriate format for the C code. However, one important part that users may be unfamiliar with is calling the C code. This involves using a variable called “tmp” (though the name of the variable is unimportant), and calling a function called “.C”. The inputs to this function are the names of the function in the C code being called, and the different input parameters required for the C code function. After calling the “.C” function, “tmp” becomes a list with each element being an input variable, though the vectors for the response time and responses (i.e., ‘tmp$rt’ and ‘tmp$resp’) will now be from the simulation, rather than the original vector of zeros.

The LUT for the LUT-iCDF method is created at the top of each R wrapper, before the main function that runs the simulation. The first line provides a value for the variable “use.interval” , which is the granularity of the LUT. This can be easily made larger or smaller as the user desires, though the results in “Assessing the LUT-iCDF approximation accuracy” should be considered before making any changes. The next line creates a sequence from 0 + *x* to 1 − *x* in increments of *x*, where *x* is granularity, and obtains the iCDF of the standard normal distribution for each of these values, which are placed in the LUT vector “use.table”. The last line obtains the size of the LUT and places it into the variable “n.table.options”.

### Worked example: simulating the LBA

Here, I provide a brief worked example of how to use the R wrappers and C code described above to simulate from the linear ballistic accumulator (LBA; Brown & Heathcote 2008). I also show how simulating from my framework differs from the recent R package *rtdists* (n All code for this worked example can be found within the “Worked-example” folder, which includes two previously discussed files (“lba.c” and “simulate-lba.R”) that are called by the example code, “lba-example.R”.

The example code (“lba-example.R”) begins by clearing the workspace on line 2. Lines 4 and 5 (currently commented out, as they are only need to be performed once) compile the C code and install the *rtdists* package, respectively. Lines 7-9 load in the necessary functions from the R wrapper and the *rtdists* package. Line 12 creates the “params” vector, which consists of named values that correspond to the LBA parameters, and can easily be changed by the user. Lines 15-18 calculate the analytic PDF for the LBA using the *rtdists* package, where line 15 creates an interval of points to obtain the density for, and lines 17 and 18 obtain the PDF for response alternatives 1 and 2, respectively. Line 21 uses the *rtdists* package to simulate 50,000 trials from the LBA, and lines 23 and 24 obtain a kernel density estimate for these simulated trials—for response alternatives 1 and 2, respectively—to compare to the analytic PDF.

Line 27 uses the “simulate.lba” function from my framework to simulate 50,000 trials from the LBA, and lines 29 and 30 obtain a kernel density estimate for these simulated trials—for response alternatives 1 and 2, respectively—to compare to the *rtdists* simulations and analytic PDF. As can be gathered from this relatively brief explanation, implementing my framework is relatively easy, and can be done within a few lines of code. Lines 34 onwards plot the densities from the *rtdists* analytic PDF, the *rtdists* simulation, and the simulation from my framework, which allows users to quickly assess how well the simulations are approximating the exact analytic density, and how well the simulations from my framework are approximating regular simulations. More precise details on the accuracy of my LUT-iCDF method can be found in “Assessing the LUT-iCDF approximation accuracy”.

### Obtaining a pseudo-likelihood function with PDA

As discussed in the introduction, some recent applications of complex EAMs with intractable PDFs have involved pseudo-likelihood methods, such as probability density approximation (PDA; Turner & Sederberg 2014; Holmes 2015). PDA involves simulating a large number of trials from the model and fitting a density kernel to these model predictions, which creates an approximate PDF for the model. Importantly, PDA allows models with intractable PDFs to be applied using state-of-the-art methods, such as Bayesian parameter estimation. A more detailed explanation of PDA can be found in Turner and Sederberg (2014) or Holmes (2015).

To help make these state-of-the-art methods more accessible for models within my framework, I have included R code that implements the full PDA process, where data and parameters are input and an approximated PDF is output. The relevant PDA file for each type of model can be found in Table 4. In each PDA file, the function “log.dens.like” performs the PDA process and is relatively simple: most input variables have been discussed previously, and the output is a single number, which is the log-likelihood of the data given the parameters. Specifically, there are four new input variables: “data”, “conds”, “bandwidth”, and “simulateFunction”. The first, “data”, is a list of three elements: “Cond”, a vector of the condition that each trial was from, “Resp”, a vector of the response alternative that was chosen for each trial, and “Time”, a vector of the response time for each trial. The second, “conds”, is a vector of the conditions used within the experiment. The third, “bandwidth”, is the bandwidth of the density kernel to be used in the PDA smoothing (Holmes 2015 recommends using Silverman’s ‘rule of thumb’; Silverman^1986^). The fourth, “simulateFunction”, is the simulation function for the model, which is the name of the main function in the R wrapper (e.g., for the regular diffusion model, this would be “simulate-DIFF”). The code within the “log.dens.like” function mostly involves re-structuring the inputs to be in the correct format for the PDA code, and looping over conditions, which the parameter values might vary between. For models with time-varying components, these components must be specified within the “log.dens.like” function.

The “log.dens.like” function calls another function in the file, “Log.likelihood.fun”, which is the PDA part of the process. The inputs for the “Log.likelihood.fun” function have each been explained previously, and the output of the function is a list. The first element of the list is itself a list, with each element of this inner list containing the approximated densities for the response times corresponding to one of the response alternatives. The second element of the main list is the number of trials that had not reached a threshold at the maximum number of time-steps (i.e., “maxCounter”; only relevant for stochastic differential equations), which can be used to penalize models and parameter sets that produce several non-terminating trials. Within the “Log.likelihood.fun” function, the first line simulates trials with the input parameter values and model simulation function. From here, the code loops over response alternatives, and performs the PDA process to obtain a PDF approximation for each response time in the data that had a response in favor of that alternative. In complex terms, this involves performing a convolution with a Gaussian filter to get an estimate of the PDF, and then using linear interpolation to obtain the density for each data point. In simple terms, this involves using R’s “density” function to obtain a PDF estimate, and then simply finding the discrete point of this function that most closely matches each data point with R’s “approx” function.

## Models

Finally, I outline all of the variants and sub-variants of EAMs that I provide simulation code for. Generally speaking, my framework covers all of the currently well-known EAMs, as well as extensions of them to time-varying drift-rate or decision thresholds. All simulation code uses the LUT-iCDF method for RNG from the normal distribution.

### The linear ballistic accumulator (LBA)

**Fig. 4:**
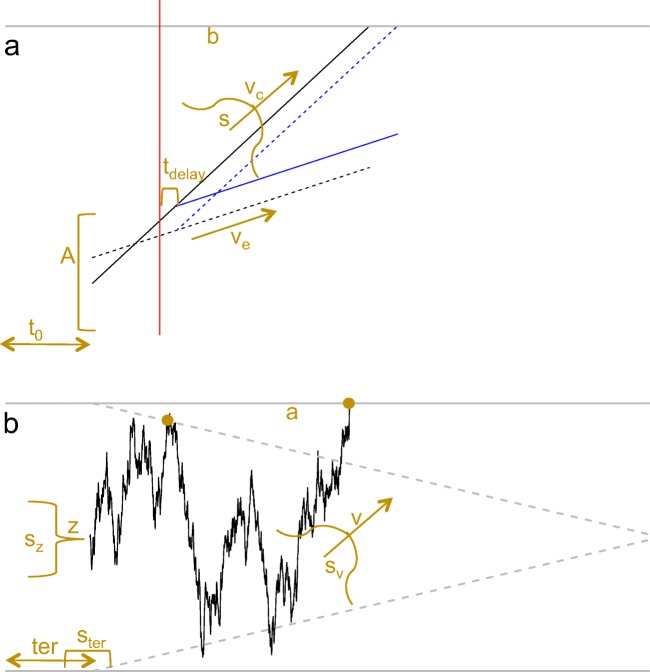
**a** An example of the accumulation process of the LBA. *Black lines* indicate the accumulation in the standard LBA for two different alternatives (*solid line* and *dashed line*). The *red line* displays a change in evidence in the stimuli, and the *blue lines* display how the accumulation for the alternatives in a piecewise extension diverge from those in the regular LBA. **b** An example of the accumulation process of the diffusion model. The *flat grey lines* display the standard fixed thresholds, and the *linearly decreasing flat lines* display a collapsing threshold with a linear collapse

The LBA is one of the simplest and most commonly applied models of rapid decision-making, which proposes a process of independent, noiseless evidence accumulation for each alternative (Fig. 4, panel a). The sources of variability within the model are in the form of a truncated normal drift rate distribution (though see Terry et al., (2015) for other potential distributions) with mean *v* and standard deviation *s* (with *s* fixed to 1 for scaling purposes, see Donkin, Brown, and Heatcote, 2009), and a uniform starting point distribution starting at 0 and ending at *A*, where *A* is always less than the threshold value *b*. The mean drift rate also differs between the alternatives, with the drift rate of the response accumulator that “matches” the stimuli (*v*_*c*_) being estimated separately from that one that “mismatches” (*v*_*e*_). In addition, the model assumes some time is dedicated to perceptual encoding and motor processes, which is labeled “non-decision time” (*t*_0_). Overall, this gives the LBA 5 general parameters: *v*, *s*, *A*, *b*, and *t*_0_.

#### Piecewise

Recent research within rapid decision-making has begun to investigate paradigms with evidence that systematically changes across a trial (e.g., (Holmes et al., 2016; Evans et al., 2017)), which fall outside of the scope of standard EAMs. (Holmes et al., 2016) proposed a “piecewise linear approximation” to account for paradigms with one or more systematic changes in evidence, in the form of the piecewise LBA (pLBA; Fig. 4, panel a). Specifically, the pLBA proposed by (Holmes et al., 2016) contains a standard LBA process up until the evidence systematically changes. After the change, the evidence is assumed to continue to accumulate unchanged for some additional amount of time, reflecting some potential delay in the uptake of new information process (*t*_*delay*_). After the delay, the drift rates for each alternative immediately change to reflect the new state of evidence. The pLBA adds three potential parameters to the standard LBA: a *t*_*delay*_ parameter, and the new mean drifts rates for the matching and mismatching accumulators after the evidence change.

### The diffusion model

The diffusion model (Ratcliff, 1978) is the most commonly applied model of rapid decision-making, which proposes a process of dependent evidence accumulation where evidence for one alternative counts as evidence against the other alternative (Fig. 4, panel b). The accumulation process is also subject to moment-to-moment noise, being the Wiener process with *σ* fixed to 0.1 (by convention) to solve a scaling issue. The simplest form of the diffusion model only contains four free parameters: the drift rate (*v*), the threshold (*a*), the starting point (*z*, which in the diffusion directly reflects response bias), and the non-decision time (*ter*). There have also been three extensions that add between-trial variability to three of the standard parameters: the drift rate, through a normal distribution with standard deviation *s*_*v*_ (Ratcliff, 1978), the starting point, through a uniform distribution with width *s*_*z*_ (Ratcliff and Rouder, 1998), and the non-decision time, through a uniform distribution with width *s*_*ter*_ (Ratcliff & Tuerlinckx, 2002). The addition of all three of these parameters is known as the “full” diffusion model, which gives the model seven general parameters: *v*, *a*, *z*, *ter*, *s*_*v*_, *s*_*z*_, and *s*_*ter*_.

#### Piecewise

The piecewise diffusion model (pDDM; Holmes & Trueblood 2018), like the pLBA, attempts to account for paradigms with systematically changing evidence via a simple piecewise linear approximation to a change in drift rate. As with the pLBA, the accumulation in the pDDM is identical to the regular diffusion until the evidence changes. After the change and some estimated delay (i.e., *t*_*delay*_), the drift rate immediately changes to reflect the new evidence, which is another estimated free parameter, resulting in an additional two free parameters in the model beyond the diffusion.

#### Time-varying drift rate

The previously discussed “piecewise” models provide a simple way of accounting for paradigms where evidence systematically varies across a trial. However, another option is to implement a stochastic differential equation with a time-varying drift rate, where instead of the drift rate being constant throughout the trial, it has the ability to differ on every time-step of the process. Time-varying models are usually constrained by the drift rate either being some transformation of the current evidence (Evans et al., 2017), or being determined by some time-varying function (Servant, Montagnini, & Burle, 2014). Time-varying models have also been previously used in situations where the evidence remains constant throughout a trial, but there is some input to directly guide how the drift rate should change over the trial, such as neural activity (Purcell et al., 2010).

#### Time-varying boundaries

One of the key assumptions of the diffusion model has been that the decision thresholds remain fixed over the course of a trial, where the same amount of evidence is required to trigger a decision regardless of the time spent on the decision. However, recent research has suggested that decision-making may involve time-varying thresholds, and more specifically collapsing thresholds, which decrease as decision time increases (Fig. 4, panel b; (Cisek, Puskas, & El-Murr, 2009; Ditterich, 2006; Drugowitsch, Moreno-Bote, Churchland, Shadlen, & Pouget, 2012; Churchland et al., 2011; Thura et al., 2012), though also see Hawkins, Forstmann, Wagenmakers, Ratcliff, & Brown, 2015). Time-varying thresholds are usually defined according to some function over time, such as the three-parameter Weibull function used by Hawkins et al., (2015) for collapsing thresholds. However, there is no consensus on a single dynamic threshold that should be applied, other than that it should decrease the threshold over time. However, where possible, it is best to use a theoretically motivated function, and the choice of the function should be defined before analysis to limit the model’s flexibility.

### The leaky-competing accumulator (LCA)

The LCA (Usher and McClelland, 2001) is one of the most complex EAMs, which was designed to be reflective of underlying neural architecture and proposes a process that contains several dependencies and non-linearities. Specifically, the LCA uses the general accumulator framework (e.g., the LBA in Fig. 4, panel a) with several added components, such as inhibition and excitation between alternatives that are estimated as a single parameter of the balance between these two processes (*β*; positive values indicate stronger inhibition). The LCA also contains a leakage component *λ*, where evidence gradually leaks away as the time from its accumulation increases, and moment-to-moment noise through the Wiener process (with *σ* fixed to 0.1, by convention, to solve a scaling issue). Overall, this gives the LCA six general parameters: three in common with the LBA (*v*, *b*, *t*_0_), and three unique parameters (*β*, *λ*, *σ*). It should also be noted that recent research has found many of the LCA parameters to show poor recovery in specific experimental paradigms, meaning that inferences made directly on the estimated parameter values may be spurious (Miletić, Turner, Forstmann, & van Maanen, 2017).

#### Piecewise

Although a piecewise LCA has not previously been implemented, I have included code to do so in the same manner as the pLBA.

#### Time-varying drift rate

As discussed for the diffusion model, time-varying drift rates provide another simple method of modeling changes in evidence. Time-varying drift rates have also been used in another situation for the LCA: paradigms where the evidence remains constant within the task, but a second source of data is used as input to drive the drift rate. Specifically, Purcell et al., (2010) used filtered single-cell recordings of monkeys from each time-step within a trial to drive the drift rate for the LCA, meaning that the drift rate was a time-varying process purely determined by neural input. However, the implementation of this model has only been performed by the original researchers, and only through basic methods (i.e., *χ*^2^), due to the computationally taxing nature of simulating this model. Using this framework, researchers familiar with EAMs can implement and test these interesting, neurally driven models using more advanced methods, provided that the neural data is made openly available for others to analyze, as previous researchers have done (e.g., Roitman and Shadlen 2002).

#### Time-varying boundaries

Like the time-varying boundaries for the diffusion model, I provide code in my framework to implement an LCA with collapsing boundaries. Interestingly, few have considered the implications of how collapsing thresholds may interact with the additional LCA components of leakage and *lateral* inhibition.

### The Urgency-Gating Model (UGM)

The UGM is a recent proposal within the rapid decision-making literature, which proposes that evidence is barely accumulated, and decisions are mostly on based upon novel input (Cisek et al., 2009; Thura et al., 2012). Specifically, the UGM takes the same basic form as the “simple” diffusion model (Fig. 4, panel b). However, in order to ensure that only novel evidence is considered, the UGM contains a low-pass filter with a time-constant (*τ*) of under 250 ms, resulting in rapid evidence leakage. In order to prevent decisions from taking too long, the evidence on each time-step is multiplied by an urgency signal (*u*) that increases with increasing time, which can be estimated as a free parameter or scale linearly with time. More specific details of this UGM implementation can be seen in Hawkins et al., (2015) and Evans et al., (2017).

#### Time-varying drift rate

As discussed for the diffusion model, time-varying drift rates provide another simple method of modelling changes in evidence. This model was also used in Evans et al., (2017).

## Conclusions

This article aimed to provide a method, framework, and tutorial for fitting complex evidence accumulation models that do not have an analytic likelihood function. Specifically, within this article I proposed a method, LUT-iCDF, for efficiently simulating decision-making models, or any type of random number generation from the normal distribution. LUT-iCDF involves using a look-up table to approximate the inverse cumulative density function method of random number generation, greatly cutting down the time taken to simulate these models, as random number generation from the normal distribution standardly can take up over 95% of the total simulation time. Importantly, I showed that LUT-iCDF with a large number of table elements closely approximates standard methods of random number generation from the normal distribution. In order to allow others to easily and efficiently implement LUT-iCDF for complex EAMs, I provided a framework that includes C and C and R code for *12* different variants of EAMs. Lastly, this article provided a detailed tutorial and worked example on how to implement the framework, which should allow researchers who are familiar with fitting simpler EAMs to extend their research to involve complex EAMs.

## Footnotes

1 My code for the standard iCDF implementation was adapted from that on John D. Cook’s website: https://www.johndcook.com/blog/normal_cdf_inverse/
